# Trends in emergency department utilization by patients with chronic conditions aged 15 and over in a tertiary-care Italian pediatric emergency department (2010–2022)

**DOI:** 10.1186/s13052-026-02209-6

**Published:** 2026-02-10

**Authors:** Veronica Casotto, Riccardo Cocco, Chiara Pipitone, Francesca Tirelli, Stefania Scanferla, Raffaella Colombatti, Silvia Bressan

**Affiliations:** 1 Department of Woman’s and Child’s Health, University of Padova, Via Giustiniani, 35128 Padova, Italy; 2 Rheumatology Unit, Department of Woman and Child Health, University Hospital of Padova, Via Giustiniani, 35128 Padova, Italy; 3 Pediatric Emergency Department, University Hospital of Padova, Via Giustiniani, 35128 Padova, Italy; 4 Pediatric Hematology-Oncology Unit, University Hospital of Padova, Via Giustiniani, 35128 Padova, Italy

**Keywords:** Chronic conditions, Adolescents and young adults, Pediatric emergency department, Health care transition

## Abstract

**Background:**

Adolescents and young adults (AYAs) with chronic conditions are increasingly surviving into adulthood, yet little is known on their use of pediatric emergency departments (PEDs). In Italy, most PEDs apply an upper age limit of 15 years, creating challenges in managing patients transitioning from pediatric to adult care. This study describes PED utilization by AYAs with chronic conditions in a tertiary care center.

**Methods:**

We conducted a retrospective study at the PED of Padua University Hospital from January 2010 to December 2022. Eligible visits included patients aged ≥ 15 years with chronic conditions. Demographic, clinical, and management data were extracted and analyzed. Trends were assessed using Joinpoint regression.

**Results:**

Overall, 2249 (0.7% of total PED visits) involved AYAs with chronic conditions. Visits increased by 241.6% from 2010 to 2011 to 2021–2022, with a significant annual growth of 18.2% after 2015. Most patients were aged 15–18 years (86.8%) and presented with acute symptoms (≥ 94%). Triage data showed 65.1% urgent and 4.4% emergent classifications. Resource use was substantial: 63.7% required laboratory tests, 25.7% imaging, 53.6% specialist consultation, and 37.6% hospital admission. Hemato-oncological diseases were the leading cause of visits, while neuropsychiatric presentations rose from 6% in 2010–2011 to 22% in 2021–2022.

**Conclusions:**

Despite institutional age limits, AYAs with chronic conditions increasingly rely on PEDs, often for complex and urgent care. Rising neuropsychiatric visits and persistent non-urgent use highlight gaps in outpatient and transitional care. Structured transition programmes and enhanced collaboration between pediatric and adult services could strengthen continuity and appropriateness of care, with recent pilot initiatives representing a promising step forward.

**Supplementary Information:**

The online version contains supplementary material available at 10.1186/s13052-026-02209-6.

## Background

In recent decades, advances in diagnosis and treatment have significantly improved the survival and life expectancy of children with chronic congenital and acquired conditions, such as cystic fibrosis, congenital heart disease, haematological and neuromuscular disorders [1–3]. As a result, an increasing number of these individuals now reach adolescence and early adulthood while still receiving care for their long-standing medical needs [4].

This growing population, referred to as Adolescents and Young Adults (AYAs) - generally defined as individuals aged 15 to 25 years - occupies a unique position in the continuum of care. AYAs often face distinct clinical, psychosocial, and organizational challenges that differ both from younger pediatric patients and from older adults. Their medical complexity, evolving autonomy, and transitional life phase require tailored approaches in both routine care and emergency settings [5].

Despite their increasing prevalence, the healthcare needs of AYAs with chronic conditions, especially within the Emergency Department (ED), remain under-explored. AYAs may present to pediatric EDs for acute exacerbations of chronic illnesses, management of disease-related complications, or due to a lack of adequate outpatient or transitional care alternatives in the adult setting [6]. However, they often exceed institutional age limits for traditional pediatric services, creating potential tension between clinical appropriateness and organizational policies [4].

Moreover, the pediatric ED may serve as a default point of contact for AYAs due to their long-standing and trusting relationships with pediatricians, limited familiarity with adult services and providers, or lack of coordinated follow-up. This can lead to suboptimal care pathways, repeated ED utilization, high readmission rates and increased healthcare resource use [7].

There is a pressing need to better understand the patterns of ED access, presenting reasons, and resource utilization among AYAs with chronic conditions who seek care in pediatric emergency settings. Data in this area are limited, especially in European contexts where age thresholds for pediatric care may vary substantially [8–10].

This study aims to provide a comprehensive description of ED utilization by AYAs with chronic conditions in a tertiary care center where the ED upper age limit is 15 years. We specifically examined visit frequency and characteristics, the clinical reasons for presentation, and associated healthcare resource use.

## Methods

### Study design and setting

A retrospective study was conducted at the Paediatric Emergency Department (PED) of Padua University Hospital, a single tertiary care center, over the period from 1 January 2010 to 31 December 2022. Eligible visits included patients aged 15 years or older. Exclusion criteria were applied at two levels. First-level exclusions comprised visits closed for administrative reasons or those in which patients left without being seen or before completing care. Second-level exclusions included visits by patients without chronic conditions, visits related to caregivers or healthcare professionals who developed acute symptoms during their stay at the PED and were subsequently referred to the adult emergency department, and visits of previously healthy patients older than15 years of age who erroneously presented to the PED.

Anonymized data were extracted from the ED visit database and included basic demographics (i.e. age groups, sex, nationality – Italian and non-Italian), clinical characteristics, and details on ED management. The clinical characteristics of these patients included: (i) the underlying reason for their ED visit, classified as acute symptoms, trauma/accident, recurrent symptoms, or other; (ii) the triage category assigned upon arrival to the ED to each patient, classified into three levels of urgency (non-urgent, urgent, and emergent); (iii) the patient’s ED medical history. The data set included information on co-payment exemptions for care of chronic diseases. The care burden experienced by patients was determined using metrics such as the number of laboratory tests, diagnostic imaging exams, and specialist consultations requested during the visit. Visit outcomes were documented as discharge to home, hospital admission, or refusal of hospital admission. Discharge diagnoses were recorded as free-text entries, along with any discharge notes. For analytical purposes, we divided the study period into consecutive two-year intervals, with the exception of the year 2020, which we considered as a standalone category due to the exceptional impact of the pandemic on emergency department activities and healthcare service utilization.

### Definition

Chronic conditions (CC) were identified through a combined analysis of disease-related co-payment exemptions, where present, and medical history-related information. Chronicity-related visits were defined as emergency department visits by patients with chronic conditions that met at least one of the following predefined criteria:

(i) a pathophysiologically plausible link between the reason for the ED visit and the underlying chronic disease;
(ii) a specialist consultation with the pediatric reference specialist for the chronic condition during the ED visit;
(iii) an ED visit related to the administration of disease-specific therapies or to malfunctions or complications of medical devices required for the management of the chronic condition;
(iv) ED access following the application of disease-specific clinical protocols recommending ED evaluation in the presence of predefined symptoms, as occurs, for example, in patients followed by pediatric onco-hematology services.

These criteria were assessed through structured review of the electronic medical records, including presenting symptoms, medical history, specialist consultations, diagnostic procedures, and treatments administered during the ED visit. Visit classification was independently performed by two investigators with clinical expertise in pediatric care; disagreements were resolved by consensus. The visits were categorized based on the pediatric specialty most relevant to each individual patient, including: Allergology/Pneumology/Clinical Immunology, Cardiology, Surgery/Urology, Endocrinology/Metabolic Diseases, Gastroenterology, Nephrology, Neurology, Child Neuropsychiatry, Hemato-Oncology, Rheumatology.

### Data analysis

Count data were expressed as numbers and proportions (%). Temporal trends in PED visits were calculated using the Joinpoint Regression software [11]. This model identifies significant changes in trends by calculating the Annual Percent Change (APC). APCs were considered statistically significant when the p-value was less than 0.05. Joinpoint regression was performed using the Joinpoint Regression Program, version 5.0.2 (National Cancer Institute). The maximum number of joinpoints allowed in the analysis was set to two, and model selection was based on the Weighted Bayesian Information Criterion (WBIC). A heteroscedastic Poisson variance was specified, as recommended for count data. Under this specification, the Joinpoint software assumes independent errors and does not allow autocorrelated errors; therefore, autocorrelation testing was not applicable. Annual Percent Change (APC) estimates and corresponding 95% confidence intervals were computed using the empirical quantile method with resampling, as implemented in the software. The analysis period was divided into seven categories: 2010–2011, 2012–2013, 2014–2015, 2016–2017, 2018–2019, 2020, 2021–2022. Statistical analyses were conducted using STATA/MP version 19.5 [12].

This retrospective study was approved by the Ethics Committee of Padua University Hospital (Approval No. 0063658/2023), which waived the requirement for informed consent due to the nature of the study.

## Results

**Fig. 1:**
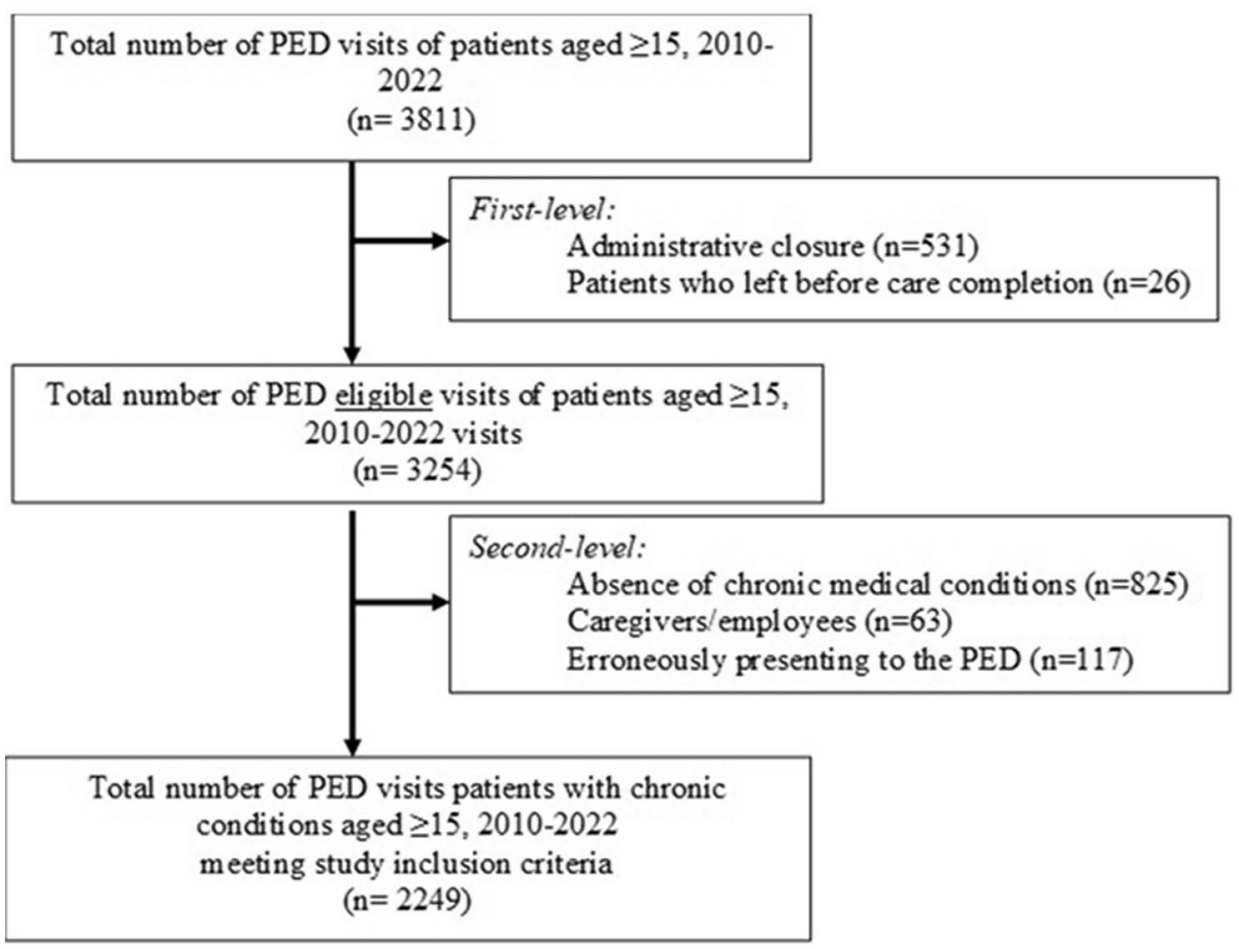
Flow-chart of pediatric emergency department visits selection and applied exclusion criteria

During the period 2010–2022, a total of 3811 PED visits of patients aged ≥ 15 was recorded out of a total of 308,411 PED visits (1.2%). Of these, 2249 visits (59%) met the study’s inclusion criteria and were analysed, of wich 87.9% were chronicity-related. The ED visits selection process is illustrated in Fig. 1, which details the application of both first- and second-level exclusion criteria.

Of the 3,254 eligible ED visits remaining after first-level exclusions, 3,074 visits were classified according to the presence or absence of a chronic condition. Among these, 2,536 visits had at least one exemption code. As a single visit could be associated with multiple exemption codes, these visits generated a total of 7,491 exemption codes, of which 4,719 were related to chronic conditions. When exemption data were unavailable or incomplete, visits were classified based on information retrieved from the medical history recorded during the ED visit (Supplementary Material, Figure S1 and Table S1).

**Fig. 2:**
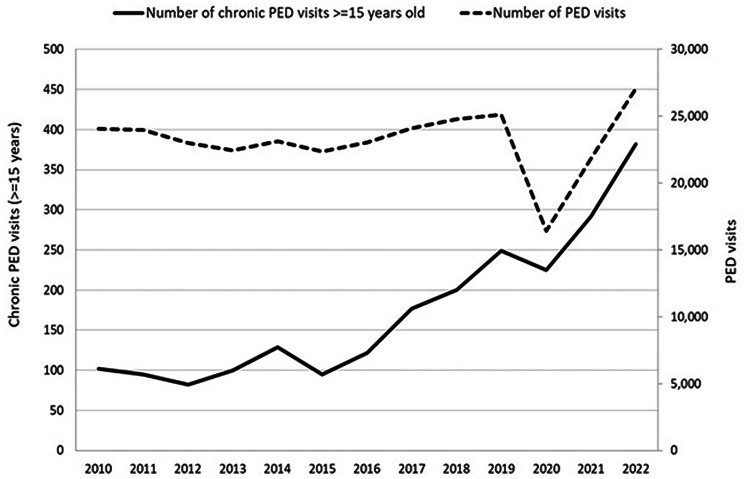
Trend of PED visits: total versus ≥ 15 years with chronic conditions, 2010–2022

During 2010–2022, the visits to the Paediatric ED of Padua University Hospital by patients aged ≥ 15 years with chronic disorders showed an increasing trend: from 197 in 2010–2011 to 673 in 2021–2022 (+ 241.6%). A statistically significant upward trend was observed from 2015 (Fig. 2). According to the results of the Joinpoint analysis, ED visits among patients aged ≥ 15 years with chronic conditions increased significantly by 18.2% per year over the 8-year period from 2015 to 2022 (*p* < 0.05). In 2020, the year of the COVID-19 pandemic, there was a decrease in ED visits by patients with chronic conditions compared to the previous year (-9.6%); however, this reduction was less marked than the overall decline in general PED visits during the same period (-34.7%).

**Fig. 3:**
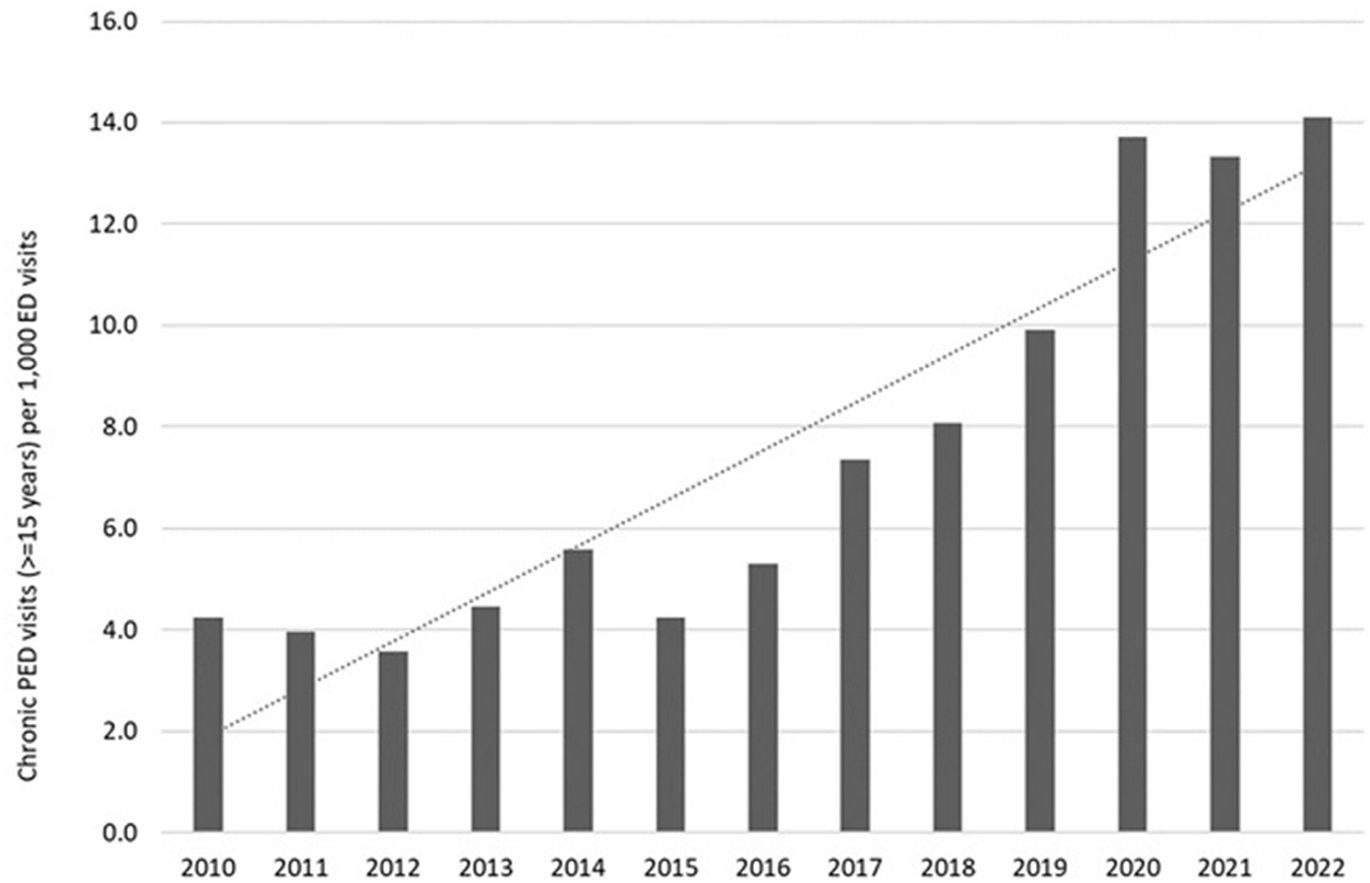
Chronic PED visits in patients ≥ 15 years per 1000 total ED visits, 2010–2022

The most significant increase in the number of PED visits among children with chronic conditions per 1000 PED visits was observed from 2020 (Fig. 3).

With respect to clinical characteristics, visits of patients aged between 15 and 18 years accounted for over 80% of visits in all periods analysed (Table 1). The percentage of visits of patients of Italian nationality increased over time, from 86.3% in the 2010–2011 period to 92.6% in the 2021–2022 period.

**Table 1:** PED visits of chronic patients ≥ 15 years by demographics and visit characteristics, 2010–2022

		2010–2011	2012–2013	2014–2015	2016–2017	2018–2019	2020	2021–2022
Total PED visits		197	182	224	299	449	225	673
***Demographic characteristics***								
Sex	Male	47.2%	54.9%	50.0%	51.2%	46.5%	53.8%	42.2%
	Female	52.8%	45.1%	50.0%	48.8%	53.5%	46.2%	57.8%
Age at admission in years	15–18	80.7%	91.8%	84.4%	89.6%	84.9%	86.2%	88.4%
	19–20	9.6%	5.5%	7.6%	7.7%	8.5%	8.4%	5.8%
	>=21	9.6%	1.1%	4.9%	2.0%	5.1%	5.3%	5.8%
Nationality	Italian	86.3%	88.5%	79.9%	83.6%	90.0%	91.6%	92.6%
	Foreign	13.7%	11.5%	20.1%	16.4%	10.0%	8.4%	7.4%
***Characteristics of PED visits***								
Chronicity-related visits		91.4%	91.8%	87.6%	90.0%	85.2%	87.7%	87.0%
Reason for arrival	Acute symptoms	94.9%	92.9%	94.6%	97.3%	94.2%	99.6%	97.6%
	Trauma/accident	0.0%	1.1%	1.3%	0.3%	3.1%	0.4%	1.6%
	Recurrent symptoms	0.5%	1.6%	0.4%	0.0%	0.2%	0.0%	0.1%
	Other	4.6%	4.4%	3.6%	2.3%	2.4%	0.0%	0.6%
Triage category	Not urgent	34.5%	28.0%	27.7%	30.8%	36.3%	33.8%	26.2%
	Urgent	59.9%	66.5%	68.3%	64.5%	60.4%	61.8%	69.5%
	Emergent	5.6%	5.5%	4.0%	4.7%	3.3%	4.4%	4.3%
Laboratory tests performed		57.9%	58.2%	63.4%	57.9%	52.8%	71.6%	74.1%
Specialist consultation		46.7%	50.5%	46.9%	51.8%	51.4%	49.8%	62.1%
Diagnostic imaging tests		24.4%	29.1%	27.2%	23.4%	26.5%	21.8%	26.6%
Outcome	Discharge	63.5%	62.6%	66.1%	60.2%	61.9%	57.3%	62.3%
	Hospital admission	36.5%	37.4%	33.9%	39.5%	37.4%	41.8%	37.0%
	Refusal of hospital admission	0.0%	0.0%	0.0%	0.3%	0.7%	0.9%	0.7%

Regarding reasons for presentation, acute symptoms predominated, accounting for over 94% of admissions across all periods and peaking at 99.6% in 2020. Triage classifications were largely urgent ranging from 59.9% to 69.5%, with a progressive decline in non-urgent cases over time. The use of laboratory tests rose from 52.8% in 2018–2019, to 74.1% in 2021–2022, accompanied by an increase in specialist consultations, which reached 62.1% in the final period. The use of imaging tests remained relatively stable, (21.8% − 29.1%). As for outcome, most patients were discharged (57.3% − 66.1%), while hospital admissions accounted for 33.9% − 41.8% of visits.

**Fig. 4:**
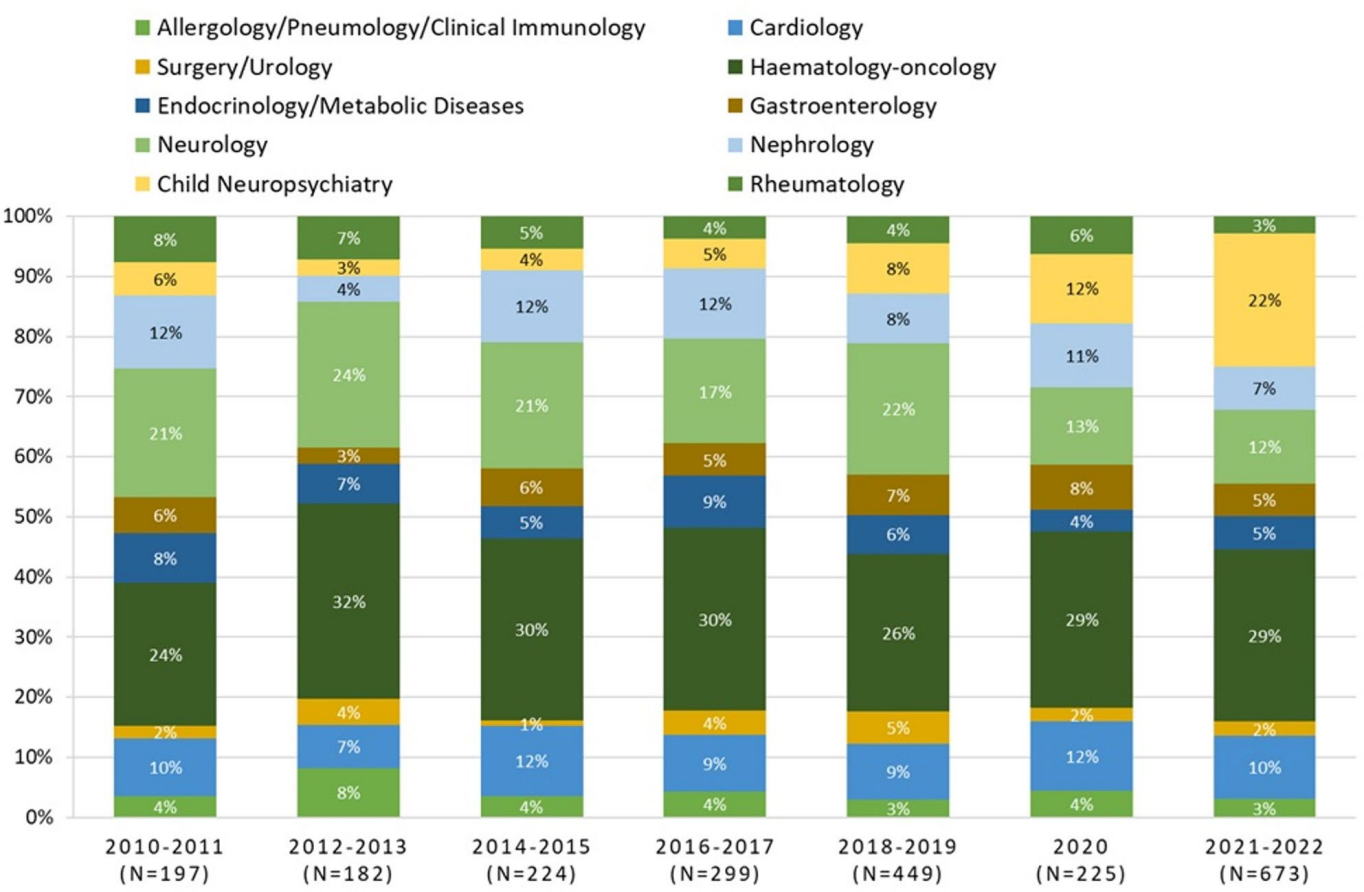
Distribution of PED visits of chronic patients ≥ 15 years by medical speciality and year, 2010–2022

Throughout the study period, haemato-oncological diseases were the leading conditions associated with ED visits among chronic patients aged ≥ 15 years (Fig. 4). Neurological disorders consistently represented the second most common cause, except in 2021–2022, when neuropsychiatric disorders ranked second. This latter group showed the largest growth over time, increasing from 6% of total visits in 2010–2011 to 22% in 2021–2022, corresponding to a rise from 11 visits in the first biennium to 149 in the last.

## Discussion

Our study addressed an underexplored area of emergency and acute paediatrics: the use of PEDs by AYAs in settings with an upper age limit of 15 years [5, 13–15]. This study provides novel insights into the emergency care utilization of AYAs with chronic conditions in tertiary-care setting. Despite the age threshold of 15 years to access the ED, a substantial and steadily increasing number of AYAs - predominantly those with congenital or childhood-onset chronic diseases - continued to access the PED over the 12-year study period. Their visits were often clinically relevant, with high rates of urgent or emergent triage classifications, extensive use of diagnostic and specialist resources, and frequent hospital admissions, underscoring the complexity of this population.

Between 2010 and 2022, visits by patients over the age of 15 accounted for 1.1% of total PED visits, consistent with prior studies [16]. However, the 413% increase over this period, highlights a clear upward trend. Approximately 70% of these visits involved patients with congenital or childhood-onset chronic diseases who continued to receive care from paediatric specialists – similar to the findings by Park et al. [5], but higher than those reported in other cohorts [14, 15]. Data from the Rare Disease Registry confirm the growing number of AYAs with childhood-onset chronic conditions in our Region ( [17]. Our findings indicate that these patients present significant challenges not only in outpatient management but also in emergency care. Data regarding the number of adolescents with chronic and/or rare disease in the catchment area (and, overall, in our Region and country) are not available due to lack of interoperability between data collection systems and registries.

The SARS-CoV-2 pandemic did not significantly reduce PED visits in this group. A temporary decline in visits occurred in 2020, during national lockdowns (March-May, October-December), largely due to reduced circulation of infectious pathogens from school closures, social distancing, and remote learning, as well as shielding measures for children with chronic conditions [18].

Several factors may explain the overall rise in emergency visits, beyond the increasing numbers of AYAs with chronic conditions. In addition, our data suggest that the increasing demand is driven by a combination of factors, including the marked rise in neuropsychiatric-related visits, the persistence of repeated emergency department use among patients with chronic conditions as they age, and heterogeneous temporal trends across pediatric specialties. First of all, the absence of structured and universally implemented transition protocols to adult services, which should encompass not only routine outpatient management but also adequate pathways of referral for the acute complications. The utilisation of paediatric emergency services by adolescents reflects their specific healthcare needs, which may be unmet by paediatricians or general practitioners. This highlights the importance of developing dedicated pathways within emergency care for adolescents and young adults, alongside structured transition programmes and stronger collaboration between pediatric and adult services. In 2022, our hospital introduced a formal transition policy with structured transition programs in several specialities, and future analyses will assess its impact on access to emergency care. Another contributing factor is the strong relational continuity with paediatric teams, which fosters prolonged reliance on familiar providers. Notably, 30% of visits were classified as non-urgent triage codes. This finding may suggest a mismatch between perceived urgency and assessed severity, highlighting potential areas for improved integration between hospital and community care within a hub-and-spoke model.

Analysis of age distribution revealed that the vast majority of visits were by patients under 18 years (86.8%), despite the local paediatric age limit being 14. The mean patient age (16.6 years) was lower than reported in other studies, typically 20–25 years [5, 15], reflecting local policy constraints. These data raise important questions regarding the potential extension of the age limit for access to paediatric emergency services. While some specialties, such as haematology-oncology, already offer continued care up to age 21 according to the disease type, expanding this policy across the board would require significant resource restructuring.

Triage data confirmed the clinical relevance of these encounters: 65.1% were classified as urgent and 4.4% as emergent. The intensity of care was also considerable: 63.7% of PED visits required laboratory tests, 25.7% involved imaging or instrumental diagnostics, and over half (53.6%) required specialist consultation. Hospital admission occurred in 37.6% of cases, consistent with previous reports [14, 19], further emphasising the complexity and acuity of this population. This underscores the need for targeted training and educational to ensure optimal care for these patients, as many adult healthcare professionals remain unfamiliar with chronic paediatric-onset conditions and their emergency management.

Specialty-specific trends in access were also identified. Haematology-oncology and neurology were the most frequently involved disciplines. AYAs with benign haematological conditions are in fact, one of the rapidly increasing group [17, 20]. Of particular concern is the steady increase in neuropsychiatric visits since 2020, in line with findings by Celona et al. [21], who reported a 322% rise over the previous decade. This trend may reflect both the exacerbation of pre-existing psychiatric conditions and the emergence of new cases linked to the psychological stress and disruption of mental health services during the pandemic. These findings underscore the urgency of integrating mental health support into emergency care, particularly for vulnerable adolescent populations.

In light of these findings, there is a pressing need to develop targeted care models for AYAs, with a deeper and broader understanding of their needs according to the type of the underlying chronic condition, the local availability of pediatric and adult specialists and the institutional health care policies and organization. These models should aim to ensure continuity of care, age-appropriate service delivery, and psychosocial support during the vulnerable phase of life that is adolescence and the transition process to adult services. Multidisciplinary collaboration between paediatric and adult care providers, both for routine outpatient health care and emergency room management, alongside community and mental health services, will be essential to address this complex challenge. Enhancing the preparedness of adult healthcare providers to manage pediatric-onset chronic diseases will be equally crucial. Ultimately, ensuring continuity, age-appropriate care, and targeted psychosocial support can improve health outcomes and reduce avoidable emergency visits during this vulnerable phase of life.

Ultimately, the importance of ensuring continuity of care, the provision of care appropriate to patients’ ages, and targeted psychosocial support cannot be overstated when considering the potential to improve health outcomes and reduce the frequency of preventable emergency visits in AYAs with chronic diseases. In the general category of chronic conditions, children who are medically complex represent a distinct subgroup, defined by their elevated care requirements and utilisation of health resources. However, the specific emergency care patterns exhibited by this group should be more thoroughly investigated within dedicated cohorts, in collaboration with pediatric palliative care services, as shown in recent Italian studies [22].

### Limitations

This study has several limitations. Its retrospective, single-centre design introduces potential selection bias and limits the generalisability of findings. The setting within a tertiary referral centre may also have resulted in an overrepresentation of complex cases. Finally, regional policies that allow paediatric care to continue up to age 18 in certain specialties may have confounded the analysis of transition dynamics, limiting comparability with other healthcare settings.

## Conclusions

Despite an institutional age limit of 15 years, AYAs with chronic conditions increasingly accessed the PED, often for urgent and resource-intensive care. The rise in neuropsychiatric presentations, of congenital disorders and persistence of non-urgent visits highlight evolving clinical needs. These findings underscore the need for structured transition programmes which include adequate emergency pathways for AYAs, strengthened collaboration between pediatric and adult services, and enhanced provider preparedness to manage pediatric-onset chronic conditions in adulthood.

## Supplementary Information

Below is the link to the electronic supplementary material.

Supplementary Material 1

## Data Availability

The datasets generated during and/or analysed during the current study are not publicly available.

## Abbreviations

AYAs: Adolescents and young adults
PED: Paediatric Emergency Department
APC: Annual Percent Change
