# Supplementary material for: Trends in emergency department utilization by patients with chronic conditions aged 15 and over in a tertiary-care Italian pediatric emergency department (2010–2022)
Source: Ital J Pediatr. 2026 Feb 10;52:41. doi: 10.1186/s13052-026-02209-6 (PMC12990577; doi:10.1186/s13052-026-02209-6)
Supplement: Supplementary file 1 — Supplementary Material 1 [file 13052_2026_2209_MOESM1_ESM.docx]

**Figure S1**. Identification of chronic ED visits



Eligible ED visits ≥15 years (2010-2022) after first-level exclusions

(n=3254)

ED visits after second-level selection exclusions

(n=3074)

ED visits that had at least one exemption code

(n=2536)

ED visits without exemption code

(n=538)

Number of exemption codes identified

(n=7491)

Exemption codes related to chronic conditions

(n=4719)

Classified using medical history review recorded during ED visit

Second-level exclusions applied for:

- Caregiver or employee visits (n=63)

- Erroneously presenting to the PED (n=117)

PED visits patient with chronic condition

(n=2249)

**Table S1.** Distribution of exemption codes of PED visits patient with chronic condition

| Code | Description | Number of ED visits | % |
| --- | --- | --- | --- |
| 3C3 | Civil disability (<18 years) | 551 | 11.7% |
| 048 | Malignant neoplastic diseases | 439 | 9.3% |
| 3C1 | Civil disability (100%) | 428 | 9.1% |
| 052 | Transplanted patients | 286 | 6.1% |
| 017 | Epilepsy | 202 | 4.3% |
| 0A0 | Heart and pulmonary circulation diseases | 196 | 4.2% |
| 007 | Asthma | 167 | 3.5% |
| A02 | Diseases of the heart and pulmonary circulation | 142 | 3.0% |
| 3C2 | Civil disability (>66%) | 137 | 2.9% |
| RDG010 | Hereditary anemias | 136 | 2.9% |
| 051 | Severe congenital physical, sensory or neuropsychiatric disabilities | 116 | 2.5% |
| 023 | Chronic kidney disease | 114 | 2.4% |
| 044 | Psychotic disorders | 103 | 2.2% |
| 013 | Diabetes mellitus | 94 | 2.0% |
| 005 | Anorexia nervosa and bulimia | 66 | 1.4% |
| RMG010 | Undifferentiated connective tissue diseases | 62 | 1.3% |
| 067 | Undifferentiated connective tissue diseases | 61 | 1.3% |
| 009 | Ulcerative colitis and Crohn’s disease | 60 | 1.3% |
| 039 | Pituitary dwarfism | 54 | 1.1% |
| 003 | Arterial hypertension with target organ damage | 46 | 1.0% |
| 027 | Congenital hypothyroidism and severe acquired hypothyroidism | 45 | 1.0% |
| 056 | Hashimoto’s thyroiditis | 45 | 1.0% |
| RCG040 | Disorders of amino acid metabolism and transport | 40 | 0.8% |
| RF0130 | Lennox–Gastaut syndrome | 40 | 0.8% |
| 040 | Premature or hospitalized neonates | 39 | 0.8% |
| 006 | Rheumatoid arthritis | 38 | 0.8% |
| 050 | Patients awaiting transplantation | 35 | 0.7% |
| 024 | Chronic respiratory failure | 32 | 0.7% |
| RNG141 | Severe and disabling congenital malformation syndromes | 31 | 0.7% |
| RNG090 | Chromosomal duplication/deletion syndromes | 30 | 0.6% |
| 016 | Chronic (active) hepatitis | 29 | 0.6% |
| RN0010 | Arnold–Chiari syndrome | 29 | 0.6% |
| 031 | Hypertensive disease (WHO stage II–III) | 27 | 0.6% |
| RCG160 | Primary immunodeficiencies | 26 | 0.6% |
| RF0040 | Rett syndrome | 26 | 0.6% |
| 059 | Celiac disease | 21 | 0.4% |
| RFG110 | Hereditary retinal dystrophies | 21 | 0.4% |
| RN1300 | Angelman syndrome | 21 | 0.4% |
| 0C0 | Diseases of arteries, arterioles, capillaries and veins | 20 | 0.4% |
| RDG031 | Chronic primary autoimmune thrombocytopenia | 18 | 0.4% |
| RN0190 | Imperforate anus | 18 | 0.4% |
| 049 | Severe multiple chronic conditions | 17 | 0.4% |
| RNG092 | Severe congenital malformation syndromes | 17 | 0.4% |
| RCG050 | Urea cycle disorders | 16 | 0.3% |
| RN0750 | Tuberous sclerosis | 16 | 0.3% |
| 0A3 | Arterial hypertension without organ damage | 15 | 0.3% |
| RN1350 | Alagille syndrome | 15 | 0.3% |
| 008 | Liver cirrhosis, biliary cirrhosis | 14 | 0.3% |
| RCG140 | Mucopolysaccharidosis | 14 | 0.3% |
| 022 | Chronic adrenal insufficiency (Addison’s disease) | 13 | 0.3% |
| RF0061 | Dravet syndrome | 13 | 0.3% |
| RF0120 | Adrenoleukodystrophy | 13 | 0.3% |
| RN0770 | Sturge–Weber syndrome | 13 | 0.3% |
| RJG020 | Primary glomerulopathies | 12 | 0.3% |
| RN0321 | Prune belly syndrome | 12 | 0.3% |
| RN1010 | Noonan syndrome | 12 | 0.3% |
| RF0140 | West syndrome | 11 | 0.2% |
| 028 | Systemic lupus erythematosus | 10 | 0.2% |
| C02 | Diseases of arteries, arterioles, capillaries, and veins | 10 | 0.2% |
| RIG010 | Progressive familial intrahepatic cholestasis | 10 | 0.2% |
| 020 | HIV infection | 9 | 0.2% |
| 021 | Heart failure (NYHA class III–IV) | 9 | 0.2% |
| RN0210 | Biliary atresia | 9 | 0.2% |
| RNG080 | Chromosomal aneuploidy syndromes | 9 | 0.2% |
| RNG121 | Severe and disabling congenital malformation syndromes | 9 | 0.2% |
| RC0220 | Antiphospholipid syndrome (primary form) | 8 | 0.2% |
| RC0241 | Familial Mediterranean fever | 8 | 0.2% |
| RN0160 | Esophageal atresia and/or tracheoesophageal fistula | 8 | 0.2% |
| RN0200 | Hirschsprung’s disease | 8 | 0.2% |
| RC0040 | Idiopathic precocious puberty | 7 | 0.1% |
| RCG150 | Chronic histiocytosis | 7 | 0.1% |
| RF0060 | Progressive myoclonic epilepsy | 7 | 0.1% |
| RN0120 | Congenital optic disc coloboma | 7 | 0.1% |
| RN0710 | MELAS syndrome | 7 | 0.1% |
| RN0950 | Kartagener syndrome | 7 | 0.1% |
| RNG100 | Other multiple congenital anomalies with intellectual disability | 7 | 0.1% |
| 003 | Acquired autoimmune hemolytic anemia | 6 | 0.1% |
| 012 | Diabetes insipidus | 6 | 0.1% |
| 035 | Graves’ disease and other forms of hyperthyroidism | 6 | 0.1% |
| 047 | Systemic sclerosis | 6 | 0.1% |
| 055 | Active pulmonary tuberculosis | 6 | 0.1% |
| RHG011 | Severe hypoventilation syndromes | 6 | 0.1% |
| RN0820 | Beckwith–Wiedemann syndrome | 6 | 0.1% |
| RN0940 | Kabuki syndrome | 6 | 0.1% |
| 018 | Cystic fibrosis | 5 | 0.1% |
| 026 | Hyperparathyroidism, hypoparathyroidism | 5 | 0.1% |
| A31 | Arterial hypertension without target organ damage | 5 | 0.1% |
| RBG010 | Neurofibromatosis | 5 | 0.1% |
| RC0243 | TRAPS syndrome | 5 | 0.1% |
| RCG060 | Disorders of carbohydrate metabolism and transport | 5 | 0.1% |
| RD0070 | Acquired aplastic anemia | 5 | 0.1% |
| RFG010 | Leukodystrophies | 5 | 0.1% |
| RFG070 | Congenital myopathies | 5 | 0.1% |
| RP0050 | Infantile apnea | 5 | 0.1% |
| 025 | Familial hypercholesterolemia (homozygous and heterozygous) | 4 | 0.1% |
| RCG030 | Autoimmune polyendocrinopathies | 4 | 0.1% |
| RD0010 | Hemolytic uremic syndrome | 4 | 0.1% |
| RDG050 | Myelodysplastic syndromes | 4 | 0.1% |
| RF0280 | Keratoconus | 4 | 0.1% |
| RF0410 | Syringomyelia / syringobulbia | 4 | 0.1% |
| RFG040 | Spinocerebellar diseases | 4 | 0.1% |
| RG0020 | Microscopic polyangiitis | 4 | 0.1% |
| RI0050 | Primary sclerosing cholangitis | 4 | 0.1% |
| RJG010 | Primary tubulopathies | 4 | 0.1% |
| RN0050 | Lissencephaly | 4 | 0.1% |
| RN1320 | Marfan syndrome | 4 | 0.1% |
| RNG060 | Congenital osteodystrophies | 4 | 0.1% |
| 030 | Sjögren’s disease | 3 | 0.1% |
| 060 | Chronic osteomyelitis | 3 | 0.1% |
| 061 | Chronic renal diseases | 3 | 0.1% |
| RB0010 | Wilms tumor | 3 | 0.1% |
| RB0020 | Retinoblastoma | 3 | 0.1% |
| RCG161 | Hereditary autoinflammatory syndromes | 3 | 0.1% |
| RD0030 | Recurrent Henoch–Schönlein purpura | 3 | 0.1% |
| RDG020 | Hereditary coagulation disorders | 3 | 0.1% |
| RFG050 | Spinal muscular atrophies | 3 | 0.1% |
| RG0010 | Rheumatic endocarditis | 3 | 0.1% |
| RM0010 | Dermatomyositis | 3 | 0.1% |
| RM0120 | Progressive systemic sclerosis | 3 | 0.1% |
| RN0090 | Axenfeld–Rieger anomaly | 3 | 0.1% |
| RNG264 | Other severe and disabling congenital malformations | 3 | 0.1% |
| RP0070 | Congenital hepatic fibrosis | 3 | 0.1% |
| 041 | Neuromyelitis optica | 2 | 0.0% |
| 0B0 | Cerebrovascular diseases | 2 | 0.0% |
| B02 | Cerebrovascular diseases | 2 | 0.0% |
| RC0150 | Wilson’s disease | 2 | 0.0% |
| RCG061 | Congenital hyperinsulinism | 2 | 0.0% |
| RCG190 | Congenital disorders of glycosylation | 2 | 0.0% |
| RFG090 | Myotonic dystrophies | 2 | 0.0% |
| RFG100 | Periodic paralyses | 2 | 0.0% |
| RG0110 | Budd–Chiari syndrome | 2 | 0.0% |
| RM0030 | Mixed connective tissue disease | 2 | 0.0% |
| RN0740 | Ivemark syndrome | 2 | 0.0% |
| RN0990 | Moebius syndrome | 2 | 0.0% |
| 038 | Parkinson’s disease and other extrapyramidal disorders | 1 | 0.0% |
| 042 | Chronic pancreatitis | 1 | 0.0% |
| 045 | Severe psoriasis (arthropathic, pustular, erythrodermic) | 1 | 0.0% |
| 046 | Multiple sclerosis | 1 | 0.0% |
| 065 | Down syndrome | 1 | 0.0% |
| RC0060 | Werner syndrome | 1 | 0.0% |
| RC0210 | Behçet’s disease | 1 | 0.0% |
| RCG072 | Congenital defects of bile acid synthesis | 1 | 0.0% |
| RCG074 | Mitochondrial oxidation disorders | 1 | 0.0% |
| RCG076 | Pyruvate metabolism disorders | 1 | 0.0% |
| RCG090 | Mucolipidosis | 1 | 0.0% |
| RD0080 | Shwachman–Diamond syndrome | 1 | 0.0% |
| RF0150 | Narcolepsy | 1 | 0.0% |
| RFG020 | Neuronal ceroid lipofuscinosis | 1 | 0.0% |
| RFG080 | Muscular dystrophies | 1 | 0.0% |
| RI0010 | Achalasia | 1 | 0.0% |
| RM0121 | SAPHO syndrome | 1 | 0.0% |
| RN0040 | Joubert syndrome | 1 | 0.0% |
| RN0330 | Ehlers–Danlos syndrome | 1 | 0.0% |
| RN0410 | Jarcho-Levis sybdrome | 1 | 0.0% |
| RN0660 | Down syndrome | 1 | 0.0% |
| RN0680 | Turner syndrome | 1 | 0.0% |
| RN0700 | Wolf–Hirschhorn syndrome | 1 | 0.0% |
| RN1270 | Williams syndrome | 1 | 0.0% |
| RN1310 | Prader–Willi syndrome | 1 | 0.0% |
| RN1330 | Fragile X syndrome | 1 | 0.0% |
| RN1360 | Alport syndrome | 1 | 0.0% |
| RN1810 | Bladder exstrophy | 1 | 0.0% |
| RNG040 | Congenital craniofacial anomalies | 1 | 0.0% |
| RNG111 | Other severe and disabling congenital malformation syndromes | 1 | 0.0% |
| Total | | 4719 | 100.0% |
